# Interaction Effects Between COVID-19 Outbreak and Fever on Mortality Among OHCA Patients Visiting Emergency Departments

**DOI:** 10.3390/medicina60122095

**Published:** 2024-12-21

**Authors:** Dahae Lee, Jung Ho Lee, Eujene Jung, Yong Soo Cho, Hyun Ho Ryu

**Affiliations:** 1 Department of Emergency Medicine, Chonnam National University Hospital, Gwangju 61468, Republic of Korea; dhlee115@naver.com (D.L.); sw97220501@naver.com (J.H.L.); em.cho.yongsoo@gmail.com (Y.S.C.); 2 Department of Emergency Medicine, Chonnam National University Medical School, Gwangju 61468, Republic of Korea

**Keywords:** COVID-19, heart arrest, fever, mortality

## Abstract

*Background and Objectives*: Fever in patients who have suffered an out-of-hospital cardiac arrest (OHCA) has been linked to poor clinical outcomes, as a fever can exacerbate neurological damage, increase metabolic demands, and trigger inflammatory responses. This study evaluates the impact of the COVID-19 outbreak and associated fevers on OHCA outcomes and examines how they can worsen patient prognosis. *Materials and Methods*: Our retrospective observational analysis used data from the National Emergency Department Information System (NEDIS), comprising adult OHCA patients at 402 EDs in Korea between 27 January and 31 December 2020 (COVID-19 pandemic period) and the corresponding period in 2019 (pre-COVID-19). The primary outcome was in-hospital mortality, with the COVID-19 outbreak as the main exposure variable and fever as an important interaction variable. We employed multilevel multivariate logistic regression with an interaction term (year of visit × fever) to examine the effects of COVID-19 and fever on mortality. Risk-adjusted mortality rates were calculated, and a difference-in-difference analysis evaluated the impact of COVID-19 on excess mortality by fever status. *Results*: During COVID-19, in-hospital mortality was higher among OHCA patients compared to the pre-pandemic period (adjusted OR 1.22, 95% CI 1.11–1.34), particularly among febrile patients (adjusted OR 1.40, 95% CI 1.24–1.59). Interaction analysis revealed that COVID-19 disproportionately increased mortality in febrile OHCA patients compared with non-febrile patients (difference-in-difference: 0.8%, 95% CI 0.2–1.5). *Conclusions*: Our study found that the COVID-19 pandemic significantly increased mortality among OHCA patients, with febrile patients experiencing disproportionately worse outcomes due to systemic delays and pandemic-related disruptions.

## 1. Introduction

Out-of-hospital cardiac arrest (OHCA) is a major global public health issue, with survival rates remaining below 10% in many regions, despite advances in resuscitation techniques [1]. For example, less than 10% of the estimated 350,000 OHCA cases annually in the United States result in survival [1,2]. In Korea, the incidence of OHCA was reported as 59.5 per 100,000 people in 2018, with rates continuing to rise. In 2019, the survival-to-discharge rate for cardiac arrest was 8.7%, with only 5.4% of patients experiencing favorable neurological outcomes [3].

COVID-19 became a global health crisis in 2020, leading to a significant rise in mortality due to acute respiratory distress syndrome and other severe complications, profoundly impacting societies and all demographics worldwide [4,5]. COVID-19 significantly increased mortality not only through direct infection but also by reducing access to healthcare services, including for OHCA patients [6,7,8]. Previous studies have reported that clinical outcomes for various conditions worsened following the COVID-19 outbreak, with this deterioration more pronounced in patients who present with fever [9,10]. Adverse clinical outcomes have been attributed to factors at both the transport stage and in the hospital [9,11,12,13,14,15].

Fever in OHCA patients is associated with poor clinical outcomes, potentially exacerbating neurological damage and increasing metabolic demands on already compromised immune systems [16,17,18]. Fever can worsen ischemic injury and lead to a cascade of inflammatory responses that may further impair recovery [19,20]. Consequently, guidelines have been developed emphasizing the importance of preventing fever in OHCA patients to mitigate secondary brain injury and improve outcomes [21].

In this study, we hypothesize that the poor prognoses of OHCA patients presenting with fever were further worsened during the COVID-19 pandemic compared to the pre-COVID-19 period, likely due to various factors affecting both pre-hospital and hospital stages. Our study aims to investigate the impact of COVID-19 and fever on OHCA outcomes and to explore the interaction between the COVID-19 outbreak and the presence of fever.

## 2. Materials and Methods

### 2.1. Study Design, Setting, and Data Sources

This retrospective observational study utilized a nationwide emergency patient database. South Korea, with a population of approximately 50 million, spans 100,210 km^2^ and includes 17 provinces and six metropolitan cities. Emergency services across the country are managed by the Korean Emergency Medical Service (EMS), a public service operated by the National Fire Agency. Nationwide, there are about 1400 ambulance stations and 219 fire stations (EMS centers). The Ministry of Health and Welfare categorizes emergency departments (EDs) into three levels according to personnel, facilities, equipment, and resource availability. Of the total 402 EDs, 38 are designated as Level 1, 125 as Level 2, and 239 as Level 3. In South Korea, Level 1 and Level 2 EDs deliver the highest level of emergency medical care services [22].

Data for this study were obtained from the National Emergency Department Information System (NEDIS), an ED-based database designed to evaluate the emergency care systems in South Korea (https://www.e-gen.or.kr/English), acessed on 2 February 2024. NEDIS contains comprehensive data on patient visits to EDs, including demographics, mode of arrival, chief complaints, visit times and dates, triage scores, diagnostic codes, dispositions, and final clinical outcomes. To protect patient privacy, data were anonymized, while accuracy was ensured through a combination of computer algorithms and manual checks [23].

### 2.2. Study Population

The study population comprised adult OHCA patients who visited any of the 402 EDs in Korea between 27 January and 31 December 2020 (COVID-19 pandemic period), and the corresponding period in 2019 (pre-COVID-19). The date 27 January 2020 was chosen as the start date for the study period as it coincided with the announcement of the “Alert” national crisis warning level for COVID-19 in Korea. The same timeframe from the previous year (2019) was used for comparison. OHCA patients were identified in the NEDIS data using the International Classification of Diseases, 10th Revision (ICD-10), with the diagnosis code I46.

### 2.3. Study Outcomes and Variables

The study outcome was in-hospital mortality at the time of discharge from the EDs or hospitals. The main exposure variable was the COVID-19 outbreak, and fever—defined as a body temperature greater than 38.0 °C upon ED arrival—was the key interaction variable. Data collected included demographic characteristics (age, sex, and residential area: metropolitan or non-metropolitan) and clinical information such as EMS use, route of ED visit, vital signs and mental status at presentation, and initial triage level as defined by the Korean Triage and Acuity Scale (KTAS).

### 2.4. Statistical Analysis

Demographic findings and outcomes are presented according to fever status and COVID-19 classification. The chi-squared test was applied for categorical variables. Using 2019 census data, incidence rates of emergency department (ED) visits per 100,000 person-days in the general population were calculated. To evaluate the COVID-19 outbreak’s impact on in-hospital mortality among ED patients, multilevel multivariate logistic regression analysis was performed, estimating adjusted odds ratios (ORs) and 95% confidence intervals (CIs) while accounting for potential confounders at both the individual and hospital levels. To analyze the interactive effects between COVID-19 and fever on study endpoints, a multilevel logistic regression model incorporating an interaction term (year of visit × fever) was used. Risk-adjusted in-hospital mortality rates and 95% CIs were subsequently estimated for each group while adjusting for variables such as year of visit, age, sex, area of residence, and clinical information (use of EMS and ED visit route) and the interaction term (year of visit × fever). The difference in risk-adjusted rates pre- and post-COVID-19 outbreak was then calculated using generalized linear mixed models to estimate excess in-hospital mortality among ED patients during the pandemic. Lastly, to determine if the effect of COVID-19 on excess in-hospital mortality varied by fever status, the difference-in-difference in risk-adjusted rates between the pre-pandemic and COVID-19 pandemic periods was calculated using a multilevel linear regression model incorporating the interaction term. All statistical analyses were performed using SAS version 9.4 (SAS Institute Inc., Cary, NC, USA). All *p*-values were two-tailed, and *p* < 0.05 was considered statistically significant.

### 2.5. Ethics Statements

The Institutional Review Board (IRB) of Chonnam National University Hospital approved this study (IRB no. 1103-153-357) on 15 January 2024.

## 3. Result

### 3.1. Demographic Findings

During the study period (January 27 to December 31 in both 2019 and 2020), a total of 4467 OHCA patients arrived at EDs in 2019 (pre-COVID-19) and 4968 arrived in 2020 (COVID-19 pandemic period). Table 1 outlines the demographic characteristics of the OHCA study patients pre-pandemic and during the COVID-19 pandemic period. Among the OHCA patients presenting at the EDs, the number with fever was 933 in the pre-COVID-19 group and 1126 in the COVID-19 pandemic period group. The ED mortality rate was significantly higher in the COVID-19 pandemic period group (76.0%) compared to the pre-COVID-19 group (72.1%) (*p* < 0.01).

Table 2 describes the demographic characteristics depending on the presence or absence of fever, showing 2059 patients with fever and 7376 without fever. The ED mortality rate was 79.4% in the fever group, significantly higher than the 72.7% rate in the non-fever group (*p* < 0.01).

### 3.2. Main Outcomes

Multilevel multivariable logistic regression analysis revealed that the adjusted OR for ED mortality in the COVID-19 pandemic period group was 1.22 (95% CI: 1.11–1.34) compared to the pre-COVID-19 group. For patients with fever, the adjusted OR was 1.40 (95% CI: 1.24–1.59) compared to those without fever (Table 3).

Interaction analysis assessed whether ED mortality during the COVID-19 outbreak differed based on fever presence, revealing adjusted ORs for the study outcome in the COVID-19 pandemic period group compared to the pre-COVID-19 group of 1.18 (95% CI: 0.95–1.47) for the non-fever group, and 1.22 (95% CI: 1.10–1.36) for the fever group (*p* < 0.01) (Table 4).

### 3.3. Difference-in-Difference Analysis

Using a multilevel multivariable linear regression model to estimate the excess in-hospital mortality among OHCA patients who arrived at the ED during the COVID-19 pandemic, the risk-adjusted ED mortality rate was higher in the COVID-19 pandemic period group (70.5%) compared to the pre-COVID-19 group (67.2%), resulting in a difference in risk-adjusted rates of 3.3% (95% CI: 1.7–5.4).

We examined the difference in the risk-adjusted ED mortality rates between the pre- and COVID-19 pandemic period groups based on fever status and found that the impact of COVID-19 on ED mortality was notably greater in the fever group than the non-fever group (difference-in-difference: 0.8% [95% CI: 0.2–1.5]) (Table 5).

## 4. Discussion

This study aimed to evaluate differences in ED mortality among OHCA patients during the COVID-19 outbreak compared to the pre-COVID-19 period and to investigate whether the presence of fever worsened this outcome. Using a nationwide emergency patient database encompassing all OHCA patients across 402 EDs, we found significantly worse clinical outcomes for OHCA patients during the COVID-19 pandemic. Additionally, an interaction effect between COVID-19 and the presence of fever was observed on ED mortality. When comparing difference-in-difference models, ED mortality in the fever group increased significantly compared to the non-fever group, suggesting that the COVID-19 outbreak negatively impacted the prognosis of OHCA patients with fever.

The COVID-19 outbreak affected healthcare systems globally, leading to challenges such as social lockdowns, shortages of medical resources and personnel, and increased health inequities [24]. These issues significantly affected the management of OHCA patients at various stages of care. In the pre-hospital phase, emergency responders faced longer response times due to the need for personal protective equipment (PPE) to mitigate virus transmission risks [25]. Additionally, selecting transport destinations for OHCA patients became more challenging due to shortages of isolation facilities and their overwhelming COVID-19 caseloads [26]. In hospitals, ED care for OHCA patients was often delayed due to COVID-19 testing protocols and stringent infection control measures [27]. These delays extended to time-sensitive procedures such as percutaneous coronary intervention (PCI), extracorporeal membrane oxygenation (ECMO), and targeted temperature management (TTM) [13,28]. Studies have documented the negative impact of these delays on patient outcomes, underscoring the challenges healthcare systems faced in delivering timely care for OHCA patients during the pandemic.

Several studies have shown that COVID-19 significantly impacted OHCA outcomes, with increases in both incidence and mortality rates during this period [6,7,8]. Factors contributing to these adverse outcomes include delays in seeking medical care due to infection concerns, reduced bystander CPR, and prolonged EMS response and transport times [3,13,29,30]. Additionally, direct complications of COVID-19, such as hypoxemia, pericarditis, arrhythmias, and thromboembolism, exacerbated OHCA severity [9,31,32,33,34]. Indirectly, fear-driven delays in seeking care for acute coronary syndromes likely contributed to the progression of myocardial ischemia to cardiac arrest, thereby worsening overall outcomes [13,35,36,37].

Similarly, our study revealed a significant increase in ED mortality among OHCA patients during the COVID-19 period. Importantly, demographic characteristics such as age, gender, and geographic distribution were similar across both periods, suggesting that systemic disruptions during the pandemic, along with COVID-19-related complications, critically contributed to increased mortality.

We also observed higher mortality in OHCA patients with fever compared to those without during both periods, which may be attributed to several mechanisms whereby fever exacerbates OHCA outcomes. Fever worsens ischemic reperfusion injury, which is triggered by the sudden restoration of circulation after cardiac arrest. This complex cascade involves the production of free radicals and other mediators that cause cerebral damage. These factors significantly contribute to anoxic brain injury, a leading cause of morbidity and mortality among resuscitated cardiac arrest patients [18,19,20,38]. Elevated body temperature (>38 °C) can arise from endogenous catecholamine release, central nervous system thermoregulatory dysfunction, or heat loss changes due to vasoconstriction. Fever has been linked to poor clinical outcomes, as it increases metabolic demands on an already compromised system, worsens ischemic reperfusion injury, and decreases recovery and survival prospects following return of spontaneous circulation (ROSC) [16,17]. Previous studies consistently highlight the detrimental effect of fever on OHCA outcomes, emphasizing its role in increasing neurological injury and overall mortality.

We also analyzed the interaction effects of fever and the COVID-19 outbreak on excess mortality in OHCA patients through a difference-in-difference model. Comparing the pre-pandemic and COVID-19 pandemic periods, OHCA patients without fever exhibited a 2.9% increase in mortality following COVID-19, whereas those with fever showed a 3.7% higher mortality rate, resulting in an excess mortality difference of 1.2% for febrile patients compared to non-febrile patients after COVID-19. This increase in mortality may be primarily attributed to prolonged EMS response and transport times during the pandemic [39,40,41,42], which have been shown in previous studies to negatively impact clinical outcomes in OHCA patients. Additionally, febrile OHCA patients often experienced delays in emergency treatment upon arrival due to the COVID-19 screening process, PPE preparation, and negative-pressure facilities for post-cardiac arrest care. These factors contributed to the excess mortality observed in febrile patients during the COVID-19 period [13,43,44].

Our study underscores the significant impact of the pandemic on OHCA patient prognoses, particularly for febrile patients. We observed a higher mortality rate in OHCA patients during the pandemic compared with the pre-pandemic period, with febrile patients experiencing disproportionately worse outcomes. Specifically, febrile OHCA patients demonstrated a 1.2% excess mortality compared to non-febrile patients. This difference is likely due to systemic delays at both the pre-hospital and in-hospital stages, including prolonged EMS response times, delayed emergency interventions, and constrained resources for post-cardiac arrest care.

These findings emphasize the critical need for healthcare systems to prepare for future health crises by addressing both the direct impacts of diseases like COVID-19 and the systemic challenges posed by pandemics in general. Emergency response systems must be strengthened to minimize pre-hospital delays, optimize transport, and ensure timely access to life-saving interventions like targeted temperature management and coronary angiography. Moreover, developing robust infection control protocols that do not compromise urgent care delivery is essential for maintaining the chain of survival for OHCA patients.

Our study has several limitations. First, since this was not a randomized controlled trial, unmeasured confounders may therefore have existed beyond the COVID-19 outbreak. Second, this study relied on retrospective data from the NEDIS database, which is primarily designed to collect information on the status, treatment, and disposition of patients visiting the emergency department. Due to the characteristics of the NEDIS database, detailed clinical variables such as underlying comorbidities or pre-hospital interventions were not available for analysis. Third, we could not determine whether OHCA patients were COVID-19-positive, which may have influenced the results, particularly for febrile patients. Fourth, unadjusted confounders during the multilevel logistic regression analysis may have affected the robustness of our findings. Fifth, we only included OHCA patients who arrived at an ED. We excluded those who did not survive pre-hospital transport or were managed exclusively in pre-hospital settings, which could have potentially introduced selection bias. Finally, our definition of “fever” relied on a single body temperature measurement upon ED arrival, which may not fully reflect the dynamic changes in body temperature during the pre-hospital and hospital phases, potentially influencing our analysis.

## 5. Conclusions

Our study highlights the significant impact of the COVID-19 pandemic on OHCA outcomes, particularly among febrile patients, who experienced disproportionately high mortality due to systemic delays in care. These findings underscore the urgent need to strengthen emergency response systems and optimize pre-hospital and in-hospital protocols. Improving these processes is crucial to enhancing OHCA outcomes and ensuring the continuity of care during future health crises.

## Figures and Tables

**Table 1:** Study population characteristics pre-pandemic and during COVID-19 pandemic period outbreak.

Variables		Total	Pre-COVID-19	COVID-19 Pandemic Period	
		No (%)	No (%)	No (%)	*p*-Value
All		9435 (100.0)	4467 (100.0)	4968 (100.0)	
Age					0.25
	20–39	598 (6.3)	270 (6.0)	328 (6.6)	
	40–59	2179 (23.1)	1048 (23.5)	1131 (22.8)	
	60–79	3919 (41.5)	1885 (42.2)	2034 (40.9)	
	80+	2739 (29.0)	1264 (28.3)	1475 (29.7)	
Gender, female		3435 (36.4)	1629 (36.5)	1806 (36.4)	0.81
Metropolis, yes		4626 (49.0)	2213 (49.5)	2413 (48.6)	0.54
ED level					0.56
	Level 1 and 2	7462 (79.1)	3544 (79.3)	3918 (78.9)	
	Other	1973 (20.9)	923 (20.7)	1050 (21.1)	
Method of visit					0.85
	EMS	8971 (95.1)	4243 (95.0)	4728 (95.2)	
	non-EMS	464 (4.9)	224 (5.0)	240 (4.8)	
Fever (BT > 38.0)					0.77
	Yes	2059 (21.8)	933 (20.9)	1126 (22.7)	
	No	7376 (78.2)	3534 (79.1)	3842 (77.3)	
Medical aid, yes		967 (10.2)	459 (10.3)	508 (10.2)	0.91
ED mortality		6994 (74.1)	3219 (72.1)	3775 (76.0)	<0.01

ED, emergency department; EMS, emergency medical service; BT, body temperature.

**Table 2:** Study population characteristics by fever.

Variables			Fever		
		Total	Yes	No	
		No (%)	No (%)	No (%)	*p*-Value
All		9435 (100.0)	2059 (100.0)	7376 (100.0)	
Period					0.24
	Pre-COVID-19	4467 (47.3)	933 (45.3)	3534 (47.9)	
	COVID-19 pandemic period	4968 (52.7)	1126 (54.7)	3842 (52.1)	
Age					0.11
	20–39	598 (6.3)	88 (4.3)	510 (6.9)	
	40–59	2179 (23.1)	406 (19.7)	1773 (24.0)	
	60–79	3919 (41.5)	906 (44.0)	3013 (40.8)	
	80+	2739 (29.0)	659 (32.0)	2080 (28.2)	
Gender, female		3435 (36.4)	772 (37.5)	2663 (36.1)	0.24
Metropolis, yes		4626 (49.0)	784 (38.1)	3842 (52.1)	<0.01
ED level					<0.01
	Level 1 and 2	7462 (79.1)	103 (5.0)	7359 (99.8)	
	Other	1973 (20.9)	1956 (95.0)	17 (0.2)	
Method of visit					
	EMS	8971 (95.1)	1910 (92.8)	7061 (95.7)	0.04
	non-EMS	464 (4.9)	149 (7.2)	315 (4.3)	
Medical aid, yes		967 (10.2)	203 (9.9)	764 (10.4)	0.24
ED mortality		6994 (74.1)	1634 (79.4)	5360 (72.7)	<0.01

ED, emergency department; EMS, emergency medical service; BT, body temperature.

**Table 3:** Multilevel multivariable logistic regression analysis on study outcomes by COVID-19 outbreak and fever.

	Total	Outcomes		Model 1			Model 2			Model 3		
	N	n	%	AOR	95% CI		AOR	95% CI		AOR	95% CI	
Period												
Pre-COVID-19	4467	3219	72.1	1.00								
COVID-19 pandemic period	4968	3775	76.0	1.24	1.14	1.37	1.23	1.12	1.35	1.22	1.11	1.34
Fever												
No	7376	5360	72.7	1.00			1.00					
Yes	2059	1634	79.4	1.38	1.23	1.56	1.38	1.22	1.55	1.40	1.24	1.59

Model 1: adjusted age and gender. Model 2: Model 1 + metropolis and medical aid. Model 3: Model 2 + method of visit and emergency department level.

**Table 4:** Interaction analysis between COVID-19 outbreak and fever on mortality of cardiac arrest patients.

	Pre-COVID				COVID-19 Pandemic Period						
	Total	Outcomes		AOR (95% CI)	Total	Outcomes					*p* for Interaction
Fever		n	%			n	%	AOR	95% CI		<0.01
No	3534	2492	70.51	Reference	3842	2868	74.65	1.18	0.95	1.47	
Yes	933	727	77.92	Reference	1126	907	80.55	1.22	1.10	1.36	

AOR, adjusted odds ratio; CI, confidence interval.

**Table 5:** Risk-adjusted in-hospital mortality rates and the differences according to the COVID-19 outbreak and fever.

	Pre-COVID-19	COVID-19 Pandemic Period	Difference (Pre vs. Post)	Difference-in-Difference
	Adjusted Rate (95% CI)	Adjusted Rate (95% CI)	Adjusted Rate (95% CI)	Adjusted Rate (95% CI)
Total	67.2% (64.5–70.0)	70.5% (67.8–73.2)	3.3% (1.7–5.4)	
Fever				
No	64.9% (61.2–66.6)	67.8% (65.1–70.5)	2.9% (1.9–4.9)	Reference
Yes	69.5% (67.0–74.1)	73.2% (70.0–76.6)	3.7% (0.1–6.4)	0.8% (0.2–1.5)

CI, confidence interval.

## Data Availability

The data of this study were obtained from the Korea Centers for Disease Control and Prevention, but restrictions apply to the availability of these data and so they are not publicly available.
